# Some Provings and Verification

**Published:** 1892-11

**Authors:** F. O. Pease

**Affiliations:** Chicato, Ill.


### SOME PROVINGS AND VERIFICATIONS.

F. O. Pease, M. D., Chicago, Ill.

*Cadmium Iodide.*—In July, 1887, I prepared by the decimal scale, the 6x potency of this salt, and during one day and evening, I took six doses.

About the only symptoms elicited were a troublesome itching of the rectum and anus, which began about the fifth day of the proving; associated with this was a constipation that proved to be obstinate and troublesome. There was frequent desire for stool, without result, or only scanty discharge of dark brown offensive stools, clay-like in consistency, and while at stool an almost irresistible desire to strain forcibly so as to push downward, or prolapse the rectum, for the relief to the itching. This itching was during the day only, and worse when walking, or heated; began about nine o'clock A. M., followed in a few moments by desire for stool, and the attempt was sure to greatly aggravate the itching while at the same time the efforts at straining seemed to relieve or were very agreeable, as also better from pressing on the partially prolapsed rectum while thus straining.

There was during the afternoon much bloating of the abdomen, with sensation of fullness, and relief from passages of flatus. This itching torture became so unbearable, and the desire to remain at stool so inconvenient, I took various remedies, but with little amelioration, and in the second week it slowly subsided, but it was a long time before my bowels regained their usual clocklike regularity.

In 1889 I cured a case of itching piles, in a carpenter, sixty-three years of age, that had withstood several remedies, he had suffered long with constipation. The case was characterized by much flatulence, which on passing started up a troublesome itching, which increased until he must go to stool, also there was aggravation of the itching when heated or when walking. While at stool the peculiarities of the above proving were closely imitated, so that I remembered my own experience. I gave him several powders of the 6x that I had used, and in a few days he reported improvement, which continued until the whole condition was removed.

Several cases of itching anus have been benefited if not cured by this remedy since. I have not repeated the proving, but will do so, and hope to develop other symptoms.

*Kali-phosphoricum.*—My proving of this drug has been incorporated by Dr. H. C. Allen in the Transactions of last year. I will report one or two cases and will say that I have been much pleased with its action in several cases.

Miss M. B. P., age twenty years, brunette, came for relief from severe headaches coming more and more frequently of late, and now almost a daily visitor. The pain is mostly in the left side from temple back to occiput, is worse from motion, or stooping, and especially worse by the noises of the school-room, when she gets "desperate."

The menses are irregular, usually too late, scanty, lasting only three days, the head pain comes on a day or two before the flow —in the morning—increasing until the second day of the flow. During the first day sharp, shooting pain in the left mastoid process going down, so severe as to make her cry out. The last period occurred seven weeks ago, scanty and short in duration; since this the headaches have been more frequent and severe. On the symptom: "Sharp shooting in the left mastoid process, going down," I gave Kali-phos.$^{1m}$, to be taken in water—four doses—and placebo every two hours until better; this was on February 10th.

March 24th.—She reported headache was better that night, but the pains in mastoid were worse or more frequent the next day, although she had better sleep that night. The menses came on the 19th, more flow than usual, and the headache with it, but the mastoid pain did not put in appearance. Is now troubled with constipation, frequent urging to stool, scanty success, passing only small pieces with a sensation, "as if much was passing" (this symptom I do not remember as belonging to the proving). I prescribed Kali-phos.$^{cm}$, one dose dry, and placebo.

April 26th.—Last two periods on time and more profuse, no headache this month, but has now some pain in lower abdomen when at stool, continuing some time after the unsuccessful effort and bloating of abdomen. Kali-phos.$^{cm}$, one dose and blanks, to report in three days if not better. Not having seen the patient since, am warranted in believing the prescription was effective.

A case of rheumatic stiffness of joints, most marked in the knees and across lumbar region. Pain in legs, worse in morning before rising, better by rubbing or gentle motion. The stiffness most marked when rising from sitting, must rock body to and fro, and take hold of something or be assisted to rise. The small of back so stiff he could not straighten up until he had been on his feet some time; walked with a shuffling step, constipation, small, dark, scanty stool after much urging, much flatulence, urine deep yellow and scanty, with reddish sediment. Kali-phos.$^{1m}$, four doses, changed the constipation and urine, and the stiffness improved gradually so that he returned to work in a few days.

*Nux-vom.*, *Carbo-veg.*—A case of peculiar dyspnœa. Was called at 4.30 A. M., found an infant of seven weeks in apparent collapse; face cold, pinched features, clammy sweat over surface, a blackish shade over temples, down sides of face and over chin and under lip, while the forehead, down each side the nose, and upper lip was waxy white, the nose blue and pointed, eyes open, pupils widely dilated, conjunctivæ dry, giving to the eyes that "fishy-eyed" expression. The muscles completely relaxed, feet and hands cold, the legs mottled, abdomen distended with gas to the utmost. Heart's action, 120, and regular (this symptom gave me courage), but the respiration was a study, and can hardly be described. A quick inspiration, the nostrils collapsing at the beginning, so that the air passed through the mouth, which opened still wider with a jerk as the nostrils collapsed, the chest expanding to its utmost, then there was an interval of two or three seconds, when the expiration occurred slower than the inspiration, and an interval of two to three seconds, and then the convulsive inspiration as before. The mouth was very dry, tongue pale, dry, and pointed—shriveled.

The child at birth was plump and apparently healthy, but the mother had suffered from "sore nipples," being treated locally with washes, etc., the last one being a solution of Bismuth, which had been used for the last ten or more days. Her milk was scanty, and being eked out with all the baby foods on the market, but baby had suffered right along with colic and constipation, physic, and peppermint, until she was much emaciated and slept but little. The colic was worse after nursing, frequent urging to pass stool, abdomen distended much of the time with gas; attending physician pronounced the case one of marasmus and hopeless, and the night before prescribed whiskey and water for the colic, injections for the constipation if the Castor Oil did not work.

At one o'clock this morning the bowels had moved after hard straining; a severe attack of colic and crying followed, which the usual dosing failed to relieve; the abdomen became very much swollen; injection of water and the soap stick did not start action. At three o'clock the child was in a state of exhaustion, and the breathing as above began.

The family physician refused to come, telephoning that he could do nothing. On the history received, I gave Nux-v., a few pellets on the tongue, dropping some milk with it, and watched the case.

The one symptom that gave me hope and courage was the heart's action, pulse "being regular" but weak and 120. Belladonna, Cuprum, Ant-tart., Opium, Phos., Carbo-veg. passed in review, but one remedy only could be given. The odor of breath was offensive, and I found "dyspnœa from flatulence, Carbo-veg." in Lippe's Repertory. This, with the general condition, determined my choice, and I placed the CM potency, a few pellets on the tongue, dropped some milk with it, and with finger on pulse, watched the case, occasionally dropping a dose of water in the open mouth. After several minutes there was a tremulous movement of the eyelids, and the pupils seemed smaller. A few moments later the tongue was moved, the color of face changed, the dark shade began to disappear, the conjunctivæ less dry, as by moving the lids there was enough moisture to brighten the eye.

From this on improvement continued, and at half-past eight I left the case for an hour, as movement had returned to the eyes and muscles, and the coldness of surface much less, and deglutition returned. The breathing was still unchanged.

At half-past nine I found the child sleeping and *breathing naturally*. The mother informed me that soon after I left the house the baby moved the head and looked around, and she took it in her arms, when it straightened out "stiff-like," and she hastily laid it down again. As she did so the breathing stopped, and she thought death had come, but she quickly saw it was natural breathing, and the little one had slept ever since. While she was telling me this, I had examined the abdomen, finding it still distended, the skin moist and warm, but my touching waked her, and this seemed to start a desire to vomit. I turned her on her side and she threw up some slimy mucus and a loud bolus of wind. Immediately after, a profuse movement of bowels and loud passage of flatus. This was the end of the distention of abdomen, and the case went on to recovery. A dose of Sulphur some days after, and now the child is a plump and healthy one.

Miss L. B. W., age eighteen; brunette; music teacher. Complained of severe backache, low down between the hips, constant burning, aching soreness, worse at night after stool, while sitting or standing, better only in a measure while lying more toward the left side, constipation for several months, desire for stool only when a large accumulation, and then putting off stool because of the misery following; stool very large, difficult to expel, requiring much effort, which exhausts her, and followed by severe pain *in back*, lasting an hour or more.

At times desire for stool, with tenesmus, resulting in discharge of a mass of thick, yellow mucus. Without going into further detail, I will state that after several days and prescriptions there was no amelioration of the backache. Graphites, Æsculus, Opium, Alumina, Silicea, Nitric Acid failed. On the afternoon of the 23d was called to her home, found her in bed, with hot, dry skin, pulse 94, temperature 101.3 F., complained of the severe pain in the back, "low down," was chilly the night before, and a restless, sleepless night followed; irritable mood, thirsty.

Described the pain as throbbing, soreness, and occasional stabbing or shooting pains up the back. Suspecting rectal trouble, I questioned closely as to the location of that pain. Placed my hand on the sacrum, she said it was lower, and still lower, on arriving at the anus she shrunk away, face and lips deadly pale, crying out: "You hurt me!"

On examination, found three ugly, inflamed fissures, the largest, in front of and to the left of mesial line, mucus membrane dark-red, hot, and dry, and in the perineal body a lump of indurated tissue, large as a hickory-nut, very sore, and in this was the throbbing, shooting pain. *Was* it an abscess or a fistula? Believed it was the former. Hepar seemed to be indicated, which I prepared in four teaspoonfuls of water, a dose to be taken every hour, or until pain was better.

At nine in the evening I called. Found there was no improvement, pain more severe, perineum much more swollen, extending forward up the left labia, patient restless, anxious, temperature 102, and decidedly cross, could not stand another such night, and she must have relief or "go gray-headed."

She described one peculiar symptom, "a heavy sensation in the tumor." Bell. and Bry. have this sensation; this, with the aggravation from motion, the constipation, thirst, cutting pain, and other Bryonia symptoms. Without further study I prepared the 30M of Bryonia in four spoonfuls water, gave a dose at twenty minutes after nine o'clock, again at twenty minutes of ten, and at ten I left my patient sleeping quietly.

Morning of 24th. She had a good night's sleep; was free from pain, but the swelling was very sore, though if she *kept still*, not much inconvenience. Left placebo, and the next day she was sitting up, dressed, and on the 28th the fissures were *healed*, the tumor in perineum was gone, there had been no discharge of pus, the bowels moved with comparative comfort on the 26th, and every day since, and the *backache* (which was not a backache), was cured.

And now (June 16th), there have been two monthly periods, without pain, and on time.

This case calls to mind another, illustrating the value of "characteristics," and of individualizing each case.

Mrs. B——, age forty-three, mother of a large family, with a history of long-continued uterine disturbance, rapid child-bearing, work and worry; frequent profuse menses; nightly attacks of asthmatic breathing; must sit up or stand up to get her breath; weary, languid, strength quickly used up, but like hundreds of overworked women, she "keeps up and around."

Was called at five o'clock in the morning; found her propped up in bed, pale and anxious face, heavy coverings about her, the room heated, hot bottles and things at her feet and legs; she had been roused at one o'clock, with a severe chill, the old asthmatic breathing, fearful that death was swiftly coming, great nervous anxiety and restlessness; the daughters did all they could to relieve her chill, but without much success.

When I arrived the chill was subsiding, and she complained of the severe pain in the third finger of the left hand, which was dark-red, and swollen to twice its usual size, up to the palm; she described the pain as being "like a red-hot coal" in the joint, or close to it, on outer side of the finger. The swelling began the day before, but she supposed it a bruise. She was restlessly moving the hand, trying to find ease, but could not in any position. I prepared Arsenicum$^{cm}$ in water, a dose every ten or fifteen minutes, until better or worse.

She took only the third dose, as the pain subsided, and she dozed off to sleep. The progress of the case from this on was interesting, because the beautiful adaptability of our law of cure was so clearly demonstrated. The paronychia progressed to suppuration, but the amount of pus was scarcely a drop or two. There was no more pain or loss of sleep; the remedies used after the Arsenicum of that morning were one dose of Hepar on the third day, and later, a dose of Silicea; and the most gratifying result in the case is that her breathing has scarcely troubled her since, and the menses have been much less in quantity, and general strength better than for years.

I would like to call attention to Gymnocladus, in inflammatory conditions of the stomach, as I have found it of value in a few cases where there was one or more of this group of symptoms, "loud belching, eructations of food some time after eating, also eructations of intensely sour water after eating, or when the vomiting comes on." *As characteristic*, and by me verified, "burning in a small round spot" in the stomach, worse after eating, but continuous. This symptom led me to the Gymnocladus, and a cure of a serious case of probably ulcer of the stomach, in a man of sixty-six years, obese and light complexion.

Following a hearty meal of boiled smoked sausage, sour potatoes, etc., a sort of Dutch lunch with beer. He was "hustled" the next morning with diarrhœa, with pain in stomach and abdomen, vomiting, and great prostration. I was not called until he had been suffering some days. The diarrhœic stools were fewer than at first, but the pain in stomach was severe, worse after eating, even the least amount, and about an hour or two after the food was eaten the vomiting began, sudden nausea, violent retching, and the ejected matter was intensely sour, making the mouth and tongue feel sore and raw; the vomiting would return every few moments, for a half-hour, and then quit for two or three hours. Great hunger immediately after each series of vomiting spells, when he *must eat* to relieve the *gnawing burning spot in stomach*, which he said he could cover with a dollar. There was also a nightly aggravation soon as he laid down, or about ten o'clock the burning was much worse, and then a spell of vomiting. Arsenicum, Carbo-veg., Argentum-nit., Iris-versicolor, Bry., Calcarea, Colchicum, Psorinum, only palliated, *i. e.*, some of them did, the patient was losing strength and courage, and I was fearful that he was nearing the end.

On the thirteenth day of the trouble (eighth day of my treatment) after a long study of the case, with Lippe's Repertory at the bedside, I selected the Gymnocladus, and gave it in water, three doses, thirty minutes apart; this was late in the night (while he was suffering the night aggravation).

He had a better season of rest, and the following morning *did not vomit after eating*, the pain was less, and the throbbing (which I have omitted giving above) was much better. The day was comfortable, no more vomiting after eating, but, while sitting up, about seven in the evening, was taken suddenly with a vomiting spell, not having time to reach the bowl, and was horrified to see a large mass of bright red blood, mixed with slime, mucus, and ingesta, thick, yellow or greenish-yellow pus, of horrible taste and odor. I was hastily telephoned for; on arriving found him sitting up in chair looking better than since his sickness. He greeted me with, "I am free from pain," and "my stomach is *better*, doctor."

I got the account of the vomiting, and examined the ejected matter as it lay on the floor. There was a double handful of ingesta mixed with blood, pus, and thick mucus. My patient made a rapid recovery from this on, and is stronger than before for three years, or since he had the rheumatism, and piles, treated under orificial philosophy.

H. M. N., age forty-two, railroad employee, weight one hundred and eighty-nine pounds, of strong physique, light hair, blue eyes, jolly disposition; came to me for relief from symptoms that had of late worried him because not understood. Vague fears of paralysis, or calamity bothered him, when suffering from pains and other symptoms.

On "taking the case" I learn:

Has always been hard worked as a boy on farm to eighteenth year, since then on railroad, out in all kinds of weather, jumping on and off cars, one-half the time on night duty. Has complained lately of pain in left shoulder worse in cold damp weather and accompanied with numbness, and "dead feeling" in the fingers, hand, and arm of that side; is anxious, and afraid of paralysis, etc. Also wanted my opinion as to the chances for *him* should he have the "bunch" on his arm cut out.

This bunch I found was a tumor, size of a base-ball, located at the posterior border of the left arm-pit, freely movable, hard, and painless, but inconvenient because of its size, interfering with lying on that side. This abnormal growth had begun about fifteen years ago, and increased slowly to its present dimensions. It was evidently a fatty tumor, and could no doubt be easily removed. Additional history as follows:

From infancy to thirteenth year had frequent attacks of croup, always brought on or worse if he played in water or got feet wet.

From fifth to thirteenth year, attacks of asthma, also brought on or aggravated by damp or cold muggy weather; better in dry warm days. Smoking cubebs, saltpetre, and much drugging had only palliated.

Many warts on hands, during childhood and youth, and frequent experience with boils, large and painful. One abscess in right groin after the annual "sheep washing" in eighteenth year.

Scabies, suppressed with Sulphur ointments, etc.

Fever and ague, in twenty-fifth or twenty-sixth year, and along with it had Quinine, Mercury, and Arsenic.

Aversion to formerly loved tobacco, smoking causes restless, hurried, "must go away" feeling; cannot stay in one place. Is forgetful, irritable, and easily moved to tears; better in cold air; can't bear hot room.

Frequent "bilious attacks," offensive mouth, and at these times aversion to and aggravation from tobacco. Of late, dusty atmosphere rouses up symptoms of the old asthma of boyhood.

Since about the time the ague was cured (?) the tumor has grown from a small nodule to its present size, never has troubled him until lately; during the last five months it has grown more rapidly than at any time in the fifteen years.

Calcarea being the closest constitutional "similar," I gave him six powders of the CM (Johnstone) potency, one to be taken each two hours that P. M., to be followed with placebo before meals and at bed-time.

This was on March 23d, on the 26th he called, saying: "Doctor, I guess I've bruised or strained that shoulder, it's awful sore and lame, pained me so I could not sleep, and I had a hard chill this morning, and sweat profusely on my back and that shoulder." Prescribed placebo, to report next morning if not better.

March 28th.—Bright and early he came; had suffered much during the night from sleeplessness, throbbing pain in the tumor, which was very sensitive to touch, could not lie on that side, chilly; worse on moving, no desire for food. On examination, found the tumor swollen, dark red, hot, and very hard, the surrounding tissues also much swollen and indurated.

I gave Hepar-s-c.$^{30}$, to be taken every two hours until the pain was relieved, then the pellets (placebo) as needed, to make him sleep.

March 30th.—The pain was relieved after the third powder, and none since; tumor still red, sore, and throbbing this morning; softening on centre, with a whitish spot showing. I allowed him to continue the flax-seed poultice, which he had put on the night before, and gave three powders of Hepar, to be taken as needed to ease the pain.

April 1st.—No pain; has taken the Hepar, good sleep, but sweats profusely on upper part of body, and is easily chilled. The tumor is softened over most of its area, central spot of skin yellow, and through it a drop of pus is oozing. I enlarged this opening with a wooden tooth-pick (which, by the way, makes a good "lance" to open boils and felons with when they are *ripe*, and do not open of themselves). With this tooth-pick I was enabled to discharge about an ounce of shreddy, offensive, curdy and "mixed" pus; the cheesy masses of matter were tough and plugged the opening repeatedly.

After this, I saw nothing of the patient for some weeks, when I learned that the suppuration continued for several days; there was no more pain or trouble, and the tumor was gone, and he has been feeling in better health and strength than for years.

I report this case to illustrate the value of adhering to the teachings of Hahnemann, as we find them in *The Organon* and *Chronic Diseases.* We are to carefully consider a case in its totality, form an image of the whole condition, and are not to prescribe until we have made a thorough study of the case from two points of view, that of the individuality of the patient, and the individuality of the most similar drug; also, we must not give any treatment to the external manifestations of disease, for these are but "vicarious" and *are not* the disease, and further, greatest weight should be given to the peculiar, striking, uncommon symptoms in the selection of the remedy, and little weight to those which come as a matter of course.

"Characteristics" of the drug or remedy must be well considered, so as to *know* the image of the remedy; while the "individuality" of the patient and his sick condition must also be most carefully studied, in relation to the *way* in which his symptoms are made characteristic by this individuality, thus enabling us to more closely meet the case by the most similar remedy.

I have but few comments to offer on these cases, only wishing to emphasize the importance of carefully individualizing each case, making a thorough study of the patient, of the characteristic symptoms of the diseased life-force, weighing well the "totality," paying least attention to those symptoms that are present (as a matter of course) because of the pathological conditions.

I believe that a marked "characteristic symptom" in the patient may carry in itself a strong image of the totality in his case, *i. e.*, the diseased state of the life-force often expresses the totality in a symptom that contains a "summing up," as it were, of the whole complex of the case, and the drug having in its pathogenesis that symptom as its individual characteristic becomes the simillimum, is individualized by that symptom, and is in accord with the law and a cure results.

Prescribing entirely by characteristics, without a careful investigation into the patient's individuality, as that individuality is expressed in and through his sick or diseased state, I fear is a frequent cause or excuse for alternation, and certainly favors the habit of routine prescribing, and hence empiricism, therefore unhomœopathic.

Each individuality has its own way of being sick, that is to say, can be sick in no other way. For example: A is suffering from typhoid fever in the only way his organism *could* be sick with that fever; his individuality stamps the disease, and the drug bearing the closest similitude to the image of his sickness becomes, under the law, the simillimum, and will cure, while B, also sick with the same, because of *his* individuality, presents an entirely different complex of symptoms, which in turn requires that remedy that corresponds to the image of B's diseased state as expressed by this individuality.

There is in each plant, drug, or substance a distinct element or essential force that makes it different from all others—the violet cannot be a cabbage, neither can one remedy do the work of another.

Early in my practice I concluded that it was not the matter, molecules, or atoms in the drug nor the quantity given, that produced the phenomena, but the force inherent within them—they were only vehicles, hence it was not a difficult matter for me to prescribe a so-called high potency, and I believe if the medical student, or the doubting Thomases, will discharge from their minds the idea that drug matter is drug force, they will more easily grasp the truth and walk into the light of real homœopathics, and realize that there is a potent force in the infinitesimals.

I would also call your attention to what I believe is needed in our colleges—that is, a better, more careful, and rational teaching of the homœopathic materia medica. How many among you assembled here who acquired their knowledge of this most distinctive, and all-important branch of our school while at college? Memorizing characteristics does not constitute a knowledge of materia medica, and the responsibility resting on the teacher of this branch is of no small proportions. The student will, of course, have his own peculiar personal ideas of medicine and its objects, and while at school, will pick up much of error and falsity that experience will probably soon remove, but prejudices and false teaching will go far toward making his judgment blind his understanding, and it seems to me, to be the duty of the professor of materia medica to see to it that his students receive only the pure gospel, according to Doctor Samuel Hahnemann and his *Organon of the Healing Art*.

There seems to be a demand for reform, and this reform must begin with the *teachers*. The preceptor has much to do with the growth in his student's mentality, of that student's future *bent* in understanding and interpretation of homœopathics—and also the professors of theory and practice, and materia medica—have much more to do with his methods of homœopathic thought and work than others in the faculty.

The teacher of materia medica should have a large latitude. The field is so broad that too many lectures can scarcely be given —provided those lectures are wise counsels, and careful instructions, pointing to a method of grasping the image of the drug, to get into the way of understanding the homœopathicity of the remedy to the disease; or to recognize the characteristic peculiarities of the patient, and apply the law of cure by also recognizing and applying the simillimum.

Hahnemann has said (*Lesser Writings*): "Medicine is a science of experiences. Its object is to eradicate disease by means of remedies. The knowledge of *remedies* and the knowledge of their employment constitute medicine."

To impart this knowledge is the duty and prerogative of the teacher in college and at the bedside. How *important* that he should do his duty *well*, and in accordance with the teachings of our Hahnemann.

